# Computed tomography of the equine caudal spine and pelvis. Pathological findings in 56 clinical cases (2018–2023)

**DOI:** 10.1111/evj.14426

**Published:** 2024-10-20

**Authors:** Nadine Kristina Elise Ogden, Katja Winderickx, John David Stack

**Affiliations:** ^1^ B&W Equine Hospital Berkeley UK; ^2^ Lingehoeve Diergeneeskunde Lienden The Netherlands; ^3^ Leahurst Equine Hospital University of Liverpool Cheshire UK

**Keywords:** CT, horse, lumbosacral, pelvis, sacroiliac

## Abstract

**Background:**

Computed tomography (CT) of the axial skeleton is increasing across many equine hospitals. CT of the pelvis and caudal spine in a large group of clinical cases has not been reported previously.

**Objective:**

To describe the pathological lesions identified in the caudal spine/pelvis in horses and ponies undergoing CT spine of this region.

**Study design:**

A retrospective case series.

**Methods:**

Horses with CT imaging of the caudal spine/pelvis were included. Horses aged under 6 months and those with CT examinations performed post‐mortem were excluded.

**Results:**

Fifty‐six horses met the inclusion criteria. Ages ranged from 8 months to 20 years and bodyweights from 85 to 680 kg (mean 488 kg). Horses presented predominantly for lameness (30/56) and poor performance (18/56). Osseous pathology was identified in 41/56 horses; including osteoarthritis of the sacroiliac joint(s) (*n*: 28), pathology of the intervertebral disc joint(s) (*n*: 12), pelvic fractures (*n*: 9), osteoarthritis of the coxofemoral joint(s) (*n*: 8), ventral spondylosis (*n*: 6), acetabular rim fracture (*n*: 2), dislocation of coxofemoral joint(s) (*n*: 2), and dorsal dermal sinus of the sacrum (*n*: 1).

**Main limitations:**

The relationship between CT imaging findings and clinical signs in horses remains unclear. Further work is required to describe the relationship between congenital, developmental, and acquired changes in the caudal spine and pelvis, and clinical signs.

**Conclusions:**

CT of the caudal spine and pelvis can be utilised in horses and ponies for diagnosing a range of clinical disorders that may be causing ‘lumbosacroiliac pain’, poor performance, hindlimb lameness, and stiffness. The pathologies we observed were diverse and many patients had multiple lesions.

## 1 INTRODUCTION

There is a growing body of work describing the computed tomographic (CT) appearance of osseous pathology in the equine caudal spine (caudal thoracic spine, lumbar spine, and sacrum) and pelvis. CT of dissected lumbar specimens has identified and differentiated both congenital, developmental, and pathological findings affecting the spinous processes, the intertransverse joints, the articular process joints, the vertebral bodies, the intervertebral discs, and the sacroiliac joints in horses.^1^, ^2^, ^3^, ^4^, ^5^, ^6^ Apart from foals^7^, ^8^, ^9^, ^10^ and a small number of Shetland ponies and miniature horses^11^, ^12^, ^13^ pathological osseous changes in the caudal spine and pelvis of live horses have not been reported on CT.

The pelvic anatomy of horses is complex and traditional imaging modalities like ultrasonography can be useful in identifying pelvic fractures,^14^ sacroiliac pathology,^15^ and coxofemoral pathology.^16^ Transrectal ultrasonography in the hands of an experienced operator can be used to effectively image parts of the ventral surfaces of the lumbosacroiliac structures including the caudomedial border of the sacroiliac joint, the ventral sacroiliac ligament, the intervertebral discs, and the lumbar and sciatic nerve roots.^17^, ^18^ The use of standing oblique radiographs of the pelvis, holds diagnostic utility in the identification of coxofemoral luxation and certain pelvic fractures, even in fully developed equines.^19^ However, radiographic quality is often limited by the size of the horse and the two‐dimensional nature of the images hampers the interpretation of this complex region.^15^, ^17^, ^19^, ^20^, ^21^, ^22^ Skeletal scintigraphy of the pelvic region has been shown to produce both false‐positive and false‐negative results when compared with clinical diagnosis of sacroiliac region pain.^23^, ^24^ In this study, we aimed to describe the pathological lesions identified in the caudal spine and pelvis in horses and ponies undergoing CT of this region.

## 2 MATERIALS AND METHODS

In this retrospective case series, horses with CT examination of the caudal spine and/or pelvis at Philip Leverhulme Equine Hospital (University of Liverpool) (Hospital 1) and Lingehoeve Diergeneeskunde (Hospital 2), between November 2018 and April 2023, were included. For this article, ‘caudal spine’ is defined as the caudal thoracic vertebrae (T16–18), the lumbar vertebra, and sacrum. Horses and ponies were included if they had part or all of the caudal spine and all or part of the pelvis included in the field of view (FOV). Horses aged under 6 months, and CT examinations that had been performed post‐mortem were excluded. Technical details pertaining to equipment, positioning, the FOVs, artefacts, and complications for these horses and ponies have been reported in a separate study.^2^

Data retrieved from the medical records included horse signalment, history, presenting signs, and diagnosis. In horses where medical records were incomplete, the missing data was recorded and reported as such. CT images were retrieved and reviewed by a board‐certified equine sports medicine and rehabilitation veterinarian (N.K.O.) or a board‐certified equine surgeon (J.D.S.) (both experienced in the interpretation of CT images in horses) using proprietary DICOM software in single and multiplanar views (RadiAnt 4.2.1 DICOM viewer or Horos v3.3.6 DICOM viewer). Reviewers were not blinded to horse signalment, history, presenting signs or diagnosis. Reviewers had access to previous imaging reports at the time of imaging revision. The medical records along with CT images were reviewed and lesion(s), if detected, were recorded for each horse.

The term ‘lumbosacroiliac region’ is used to describe the caudal lumbar spine, sacrum, and articulations between the sacrum and ilium. Lesions were classified into the following categories by broad anatomical location (1–3) and subcategories based on more specific anatomical location and the type of lesion (a–d):

1. Lumbosacroiliac region (LSI)
  - Osteoarthritis of the sacroiliac joint(s)
  - Pathology of the intervertebral disc joint(s)
  - Ventral spondylosis
  - Dorsal dermal sinus
2. Coxofemoral joint
  - Osteoarthritis of the coxofemoral joint(s)
  - Acetabular rim fracture(s)
  - Dislocation of coxofemoral joint(s)
3. Pelvic fracture
  - *Tuber ischium* fracture(s)
  - *Tuber coxae* fracture(s)
  - Other pelvic fracture(s)

Lesions were defined as ‘present’ or ‘absent’. Indiscernible or uncertain lesions were excluded. If a horse had more than one lesion, each lesion was recorded. Osteoarthritis of the coxofemoral joint(s) and sacroiliac joint(s) were graded as ‘mild’, ‘moderate’, or ‘severe’ using a modified Kellgren and Lawrence grading scale for osteoarthritis.^25^ This scale was modified by grouping Grade 1 (doubtful joint space narrowing or possible lipping) and Grade 0 (normal joint) as ‘normal’. Joints graded as ‘mild’ osteoarthritis corresponded to Kellgren and Lawrence Grade 2 (definite osteophytes, possible joint space narrowing), and ‘moderate’ osteoarthritis corresponded to Grade 3 (moderate osteophytes, definite joint space narrowing, some sclerosis, possible bone‐end deformity) and ‘severe’ to Grade 4 (large osteophytes, marked joint space narrowing, severe sclerosis, definite bone ends deformity).^25^

Acetabular rim fracture, dislocation of coxofemoral joint, and pelvic fractures were graded as ‘present’ or ‘absent’. Fractures were described as ‘chronic’ or ‘acute’ based on imaging findings and clinical history.

## 3 DATA ANALYSIS

Descriptive statistics were used to analyse clinical and imaging findings using IBM SPSS Statistics Version 29.0.1.0 (171). Confidence intervals (CI) were calculated for proportions using Exact (Binominal) Symmetrical 95% CI.

## 4 RESULTS

### 4.1 Signalment

Sixty‐five horses with CT studies of the caudal spine and/or pelvis were retrieved out of which 56 horses met the inclusion criteria and were included in the study. Four horses did not meet the inclusion criteria based on age (under 6 months of age) and five horses based on the CT being performed post‐mortem. Out of the 56 horses included in the study, 13 horses were from Hospital 1, and 43 horses were from Hospital 2. The signalment and diagnosis for included horses are summarised in Table S1.

Ages ranged from 8 months to 20 years (mean 8.4 years, median 7 years). The bodyweights were between 85 and 680 kg (mean 488 kg, median 528 kg). Warmblood or warmblood crosses (*n*: 28) were most common. Other horse breeds included Friesian (*n*: 6), Thoroughbred (*n*: 3), cob (*n*: 2), Appaloosa (*n*: 2), Standardbred (*n*: 1), Andalusian (*n*: 1), Irish Hunter (*n*: 1) and Arabian (*n*: 1). Pony breeds included Miniature horse (*n*: 2), Welsh section A and B (*n*: 2), Icelandic horse (*n*: 2), Shetland pony (*n*: 2), New Forest pony (*n*: 1), unknown pony breed (*n*: 1), and Highland pony (*n*: 1). Male horses (37/56); geldings (30/56); and colts/stallions (7/56), were more prevalent than mares/fillies (19/56).

### 4.2 Presenting signs, history, and indications for CT

Horses were presented predominantly for lameness (30/56, 53.6%, CI 39.7%–67.0%) and poor performance (18/56, 32.1%, CI 20.3%–46.0%) (including difficulty with collection, lack of impulsion, difficulty with flying changes, inability to lunge in one direction, biting toward hind quarters when not ridden and bolting under saddle, bucking, difficulty backing up, wide hindlimb gait, and poor‐quality canter or difficulty maintaining canter in one direction). A history of trauma and/or clinical signs of trauma was present in a quarter of horses (14/56, 25.0%, CI 14.4%–38.4%).

Eleven horses (case number 9–19) (11/30, 36.7%, CI 19.9%–56.1%) underwent CT to investigate lameness following localising ultrasound findings. Out of these, four horses had abnormalities detected on ultrasound examination of the coxofemoral joints, two had abnormalities of both coxofemoral joints and lumbosacroiliac region, two had abnormalities of the lumbosacroiliac region, one had abnormalities of the lumbar articular process joint(s) (APJ) and the proximal suspensory ligaments, one had abnormalities of the coxofemoral joint and suspensory ligament branch, and one had abnormalities of the stifle. CT was performed in six horses (case number 1–6) (6/30, 20.0%, CI 7.7%–38.6%) to investigate lameness that had not been able to be localised using diagnostic anaesthesia, radiography, and ultrasonography. Four horses (case number 20–23) (4/30, 13.3%, 3.8%–30.7%) had CT to investigate lameness following the detection of scintigraphic abnormalities in the pelvic region; one horse was diagnosed with a *tuber coxa* fracture 8 months earlier following scintigraphy, one had increased radiopharmaceutical uptake (IRU) of the *tuber ischium* consistent with fracture, and two had IRU of the coxofemoral joint (the two horses with IRU of the coxofemoral joints on scintigraphy were not diagnosed with pathology relating to this joint on CT). Two horses (case numbers 27–28) (2/30, 6.7%, CI 0.8%–22.1%) with lameness were referred for imaging: one horse was referred for CT based on the referring veterinarian suspecting a *tuber ischium* fracture and one horse for lameness suspected to be associated with the stifle and lumbosacroiliac region (CT of the pelvis and stifle were performed). One horse (case 7) (1/30, 3.3%, CI 0.1%–17.2%) had CT to investigate lameness that had been too mild to investigate with diagnostic anaesthesia and had not been able to be localised using radiography, and ultrasonography. One horse (case 8) (1/30, 3.3%, CI 0.1%–17.2%) had CT to investigate lameness that had previously been localised to the coxofemoral joint with intra‐articular diagnostic anaesthesia. After treatment for proximal suspensory desmitis, one horse (case 29) (1/30, 3.3%, CI 0.1%–17.2%) had a CT scan of the pelvis and affected hindlimb due to failure of clinical signs to resolve. One horse (case 30) (1/30, 3.3%, CI 0.1%–17.2%) presented for investigation of lameness and was diagnosed with phlebitis of the saphenous vein (incomplete records citing reason for CT) (Table S1).

Ten horses (cases 31–40) (10/17) presented with poor performance, had CT based on localising ultrasound findings: five horses had abnormalities of the lumbosacroiliac region (*transrectal* ultrasound), one horse had abnormalities of the lumbosacroiliac region (*transrectal* ultrasound) and cervical spine, one horse had abnormalities of the lumbosacroiliac region (*transrectal* ultrasound), *tuber ischium* and cervical spine, one horse had abnormalities of the lumbar APJs and cervical spine, one horse had abnormalities of the lumbosacroiliac region (*transrectal* ultrasound) and the coxofemoral joint, and one had abnormalities of the lumbosacroiliac region (*transrectal* ultrasound) and the stifle. Four cases (cases 44–47) (4/17) had CT of the caudal spine/pelvis based on clinical suspicion of lumbosacroiliac pain. Three horses (cases 41–43) (3/17 had CT to further investigate poor performance following scintigraphic findings): one horse had IRU of the coxofemoral joint and sacroiliac joint region, one horse had IRU of the sacroiliac joint region and one horse had IRU of the coxofemoral joints and of the dorsal spinous processes of the thoracic spine. One case (case 48) (1/17) was referred for imaging of this region to investigate poor performance (Table S1).

Four horses presented for asymmetry of the pelvic region (cases 49–52) (4/56, 7.1%, CI 2.0%–17.3%) (including focal muscle atrophy of the biceps femoris, muscle atrophy of the gluteal muscles, asymmetric tail carriage, and asymmetrical croup), and four horses (cases 53–56) (4/56, 7.1%, CI 2.0%–17.3%) were presented for other reasons: chronic discharging tract over the caudodorsal sacrum, muscle atrophy of the gluteal muscles post‐foaling, crouched hindlimb gait following a traumatic incident as a foal, and one horse presented for imaging with unclear records relating to presenting complaint (Table S1).

### 4.3 Type and distribution of lesions

**FIGURE 1:**
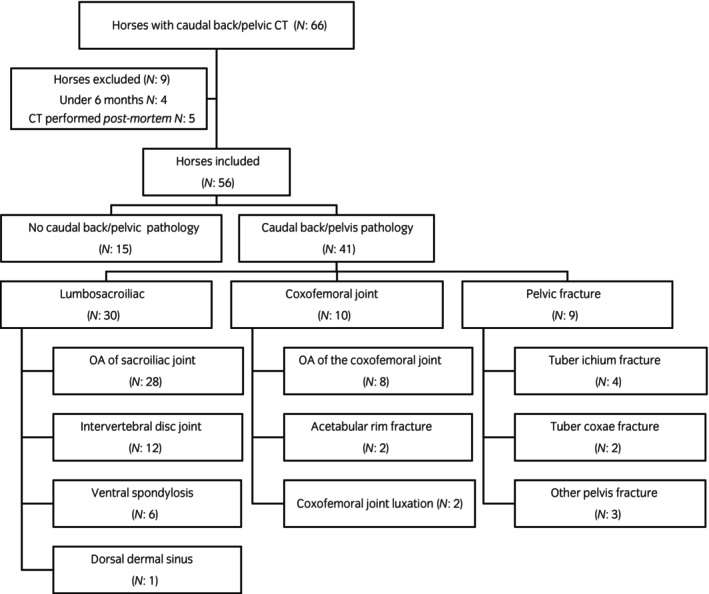
Flowchart outlining case inclusion and lesions categorisation. Horses with more than one lesion were included in each relevant broad anatomical location and subcategory based on more specific anatomical location and the type of lesion.

Osseous lesions of the caudal spine/pelvis were identified in 41 horses (41/56, 73.2%, CI 59.7%–84.2%) following caudal spine/pelvic CT, with no lesions detected in the remaining horses (Figure 1 and Table S1). Lesions were most common in the lumbosacroiliac region, followed by lesions of the coxofemoral joint(s) and pelvic fractures (Figure 1 and Table S1). In the remaining 15 horses, CT did not reveal any osseous caudal spine/pelvis pathology. In four of these cases, a diagnosis was not reached.

Seven horses (7/56; 12.5%, CI 5.2%–24.1%) had lesions identified in more than one category (lumbosacroiliac, coxofemoral joints, and pelvic fracture) (Table S1). Specifically, three horses had lesions of the lumbosacroiliac region as well as pelvic fractures, one horse had lesions of the lumbosacroiliac region, coxofemoral joint and pelvic fracture, two horses had concurrent lesions of the lumbosacroiliac region and coxofemoral joints, and one horse had concurrent lesions of the coxofemoral joints and pelvic fracture (bilateral coxofemoral joint osteoarthritis and chronic unilateral *tuber ischial* fracture).

#### 4.3.1 Pathology of the lumbosacroiliac region

**FIGURE 2:**
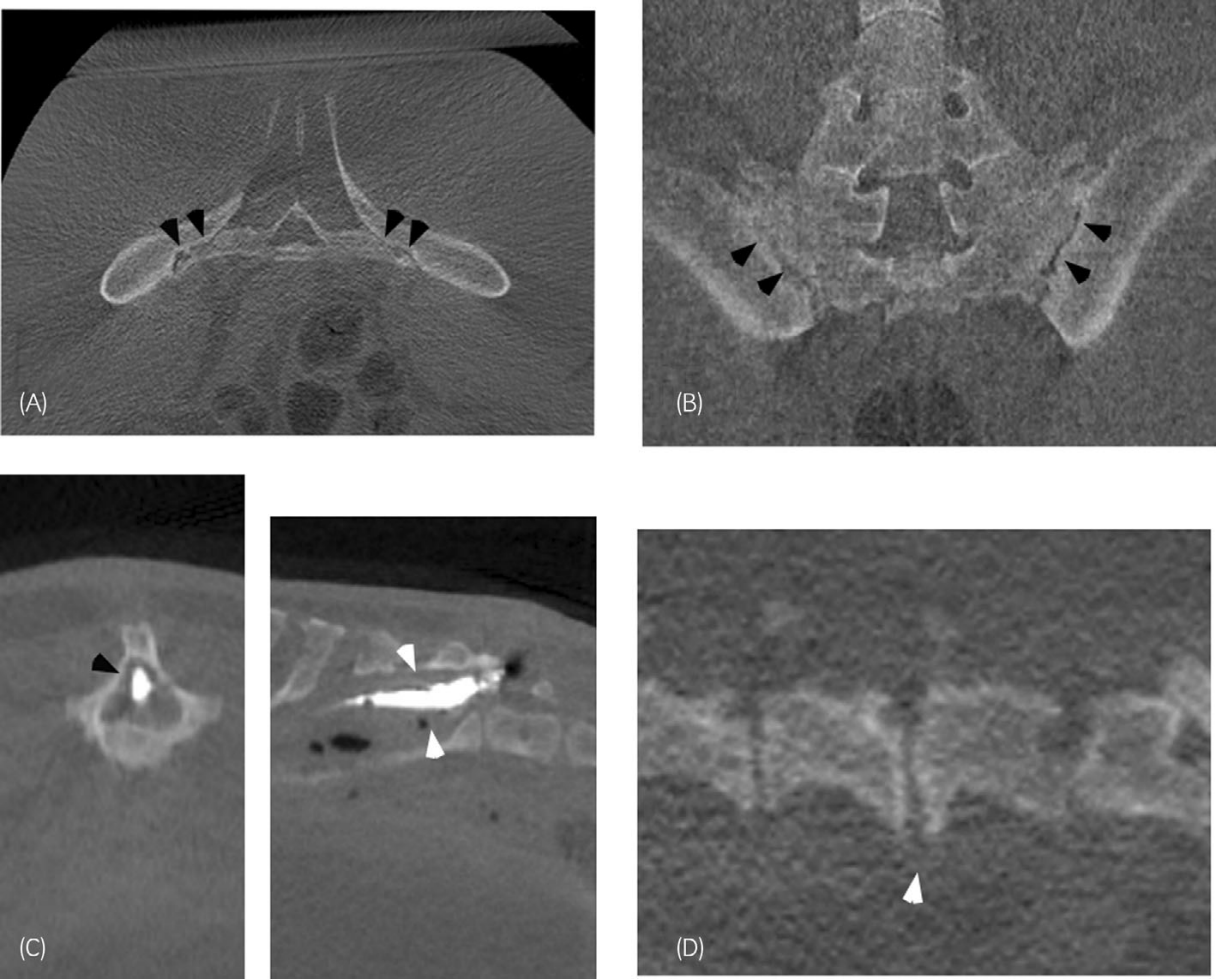
CT images (in bone window) of pathology in the lumbosacral region. Left is to the right in transverse and frontal images and cranial is to the left in sagittal images. Transverse (A) and frontal (B) images centred at the sacroiliac joint in the same horse demonstrating an irregular sacroiliac joint (black arrowheads). A transverse (left) and a sagittal (right) image (C) centred over a sacrum demonstrating contrast material within the tract of the horse with a dorsal dermal sinus (white arrowheads). A sagittal image (D) centred over the caudal lumbar spine showing non‐bridging ventral spondylosis (white arrowhead).

Thirty horses had lesions in the lumbosacroiliac region, of which, osteoarthritis of the sacroiliac joint(s) (1a) was most common, followed by pathology of the intervertebral disc joint(s) (1b), ventral spondylosis (1c), and dorsal dermal sinus (1d) (Figure 2).

### 4.4 Osteoarthritis of the sacroiliac joint(s)

Osteoarthritis of the sacroiliac joint(s) was present in 28 horses (3–20 years, median 10.5 and mean 10.0 years) with imaging findings including joint space asymmetry with regions of uneven widening and narrowing and sites with loss of distinct joint space, osseous resorption and sclerosis of the subchondral and/or trabecular periarticular bone, and periarticular new bone (osteophytes) (Figure 2). Osteoarthritis of the sacroiliac joints was more common bilaterally (22/28; 78.6%, CI 5.19%–91.7%) than unilaterally (6/28; 21.4%, CI 8.3%–41.0%). All unilateral cases had mild osteoarthritis (three were left‐sided and three were right‐sided). Out of the bilateral cases, 1 had severe bilateral OA, 15 had moderate bilateral OA, 4 had mild bilateral OA, and 2 horses had moderate OA on the left side and mild on the right side.

### 4.5 Pathology of the intervertebral disc joint

Imaging findings of the intervertebral disc joint of the lumbar or lumbosacral joint included narrowing or complete loss of the disc space, sclerosis, and/or lysis of the cranial or caudal end plates of the vertebral body, buttressing of the cranial or caudal end plates of the vertebral body, mineralisation within the lumbosacral intervertebral disc joint, impingement of the ventral lumbosacral spinal foramina, and subluxation of the lumbosacral intervertebral disc joint (Figure 2).

Pathology was present in the lumbar and/or lumbosacral intervertebral disc joint(s) in 12 horses (3–17 years, median 10 and mean 9.8 years). Of these 12 horses, pathology was only affecting the lumbosacral intervertebral disc joint in five horses (5/12) and only one lumbar intervertebral disc joint (L5–6) in one horse (1/12). Pathology of the lumbosacral disc and the intervertebral disc joint between L4 and L5 was present in two horses (2/12). Pathology of the lumbosacral disc and the intervertebral disc joint between L5 and L6 was present in two horses (2/12). Pathology affecting all the intervertebral disc joint between L4 and L6, as well as the lumbosacral intervertebral disc joint, was present in two horses (2/12).

### 4.6 Ventral spondylosis

Ventral spondylosis was seen as new bone formation abaxial to the midline at the caudal or cranial end plate of the vertebral body, partially bridging the interverbal disc joint. Non‐bridging ventral spondylosis was present in six horses (4–16 years, median 13 and mean 11.17 years) at L4–L5 (Figure 2).

### 4.7 Dorsal dermal sinus

One horse (10 years) had a chronic (years) history of a draining tract over the caudodorsal aspect of the sacrum with no history of trauma. CT revealed a gas‐filled tract extending from the dorsal skin ventrally and cranially, entering the spinal canal between the last sacral vertebra and the first coccygeal vertebra, compatible with a dorsal dermal sinus (Figure 2). A midline defect was present in the last sacral dorsal spinous process.

#### 4.7.1 Coxofemoral joint

**FIGURE 3:**
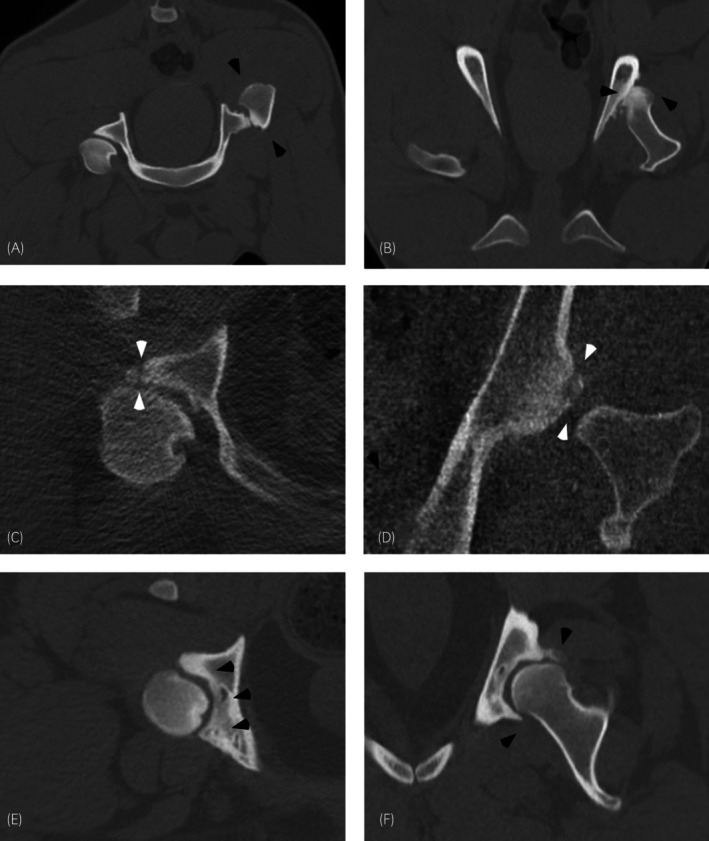
CT images (in bone window) of three horses demonstrating pathology of the coxofemoral region. Left is to the right in transverse and frontal images. Transverse (A) and frontal (B) images centred over the femoral head in the same pony demonstrating a cranially displaced dislocated femoral head (black arrowheads). Note the new bone formation along the left ilium at the dislocated femoral head. Transverse image (C) and frontal images (D) centred over the left coxofemoral joint in the same horse demonstrating an acetabular rim fracture (white arrowheads). Transverse image (E) and frontal images (F) centred over the left coxofemoral joint in the same pony demonstrating marked osteoarthritis (black arrowheads).

Ten horses (10/56; 17.9%, CI 8.9%–30.4%) had changes associated with the coxofemoral joint, with the most common lesion being osteoarthritis of the coxofemoral joint(s) (2a), followed by acetabular rim fractures (2b) and coxofemoral luxation (Figure 3). Both horses with acetabular rim fractures (2b) also had coxofemoral joint osteoarthritis (2a). There was a history of trauma in half of the horses (4/10), including both ponies with coxofemoral luxation.

### 4.8 Osteoarthritis of the coxofemoral joint

Osteoarthritis of the coxofemoral joint(s) was present in eight horses (5–20 years, median 7.5 years and mean 9.1 years) ranging from mild to severe. Osteoarthritis was more common bilaterally (5/8) than unilaterally (3/8). Four horses (4/8), with bilateral mild to moderate osteoarthritis, presented for poor performance as the primary concern. Three horses (3/8), with unilateral mild to severe osteoarthritis, presented for investigation of lameness of the affected limb. One horse (1/8) presented for asymmetry of the *tuber coxae* and was diagnosed with both osteoarthritis of the sacroiliac joints and a left *tuber coxae* fracture, in addition to severe osteoarthritis of the left coxofemoral joint (and mild osteoarthritis of the right coxofemoral joint).

### 4.9 Acetabular rim fractures

Acetabular fractures were diagnosed in two horses (2/10) (8 and 20 years) with concurrent coxofemoral joint osteoarthritis (2a); one horse had a unilateral fracture with marked osteoarthritis of the affected side and mild osteoarthritis of the contralateral limb (as well as moderate bilateral osteoarthritis of sacroiliac joints). The other horse had bilateral acetabular rim fractures and bilateral osteoarthritis of the coxofemoral joints.

### 4.10 Coxofemoral joint dislocation

Two small horses (Shetland pony and Miniature horse, 4 and 6 years) had traumatic luxation of the left coxofemoral joint with no signs of hip dysplasia. In both horses, CT was performed before surgical treatment (unilateral total hip arthroplasty).

#### 4.10.1 Pelvic fractures

**FIGURE 4:**
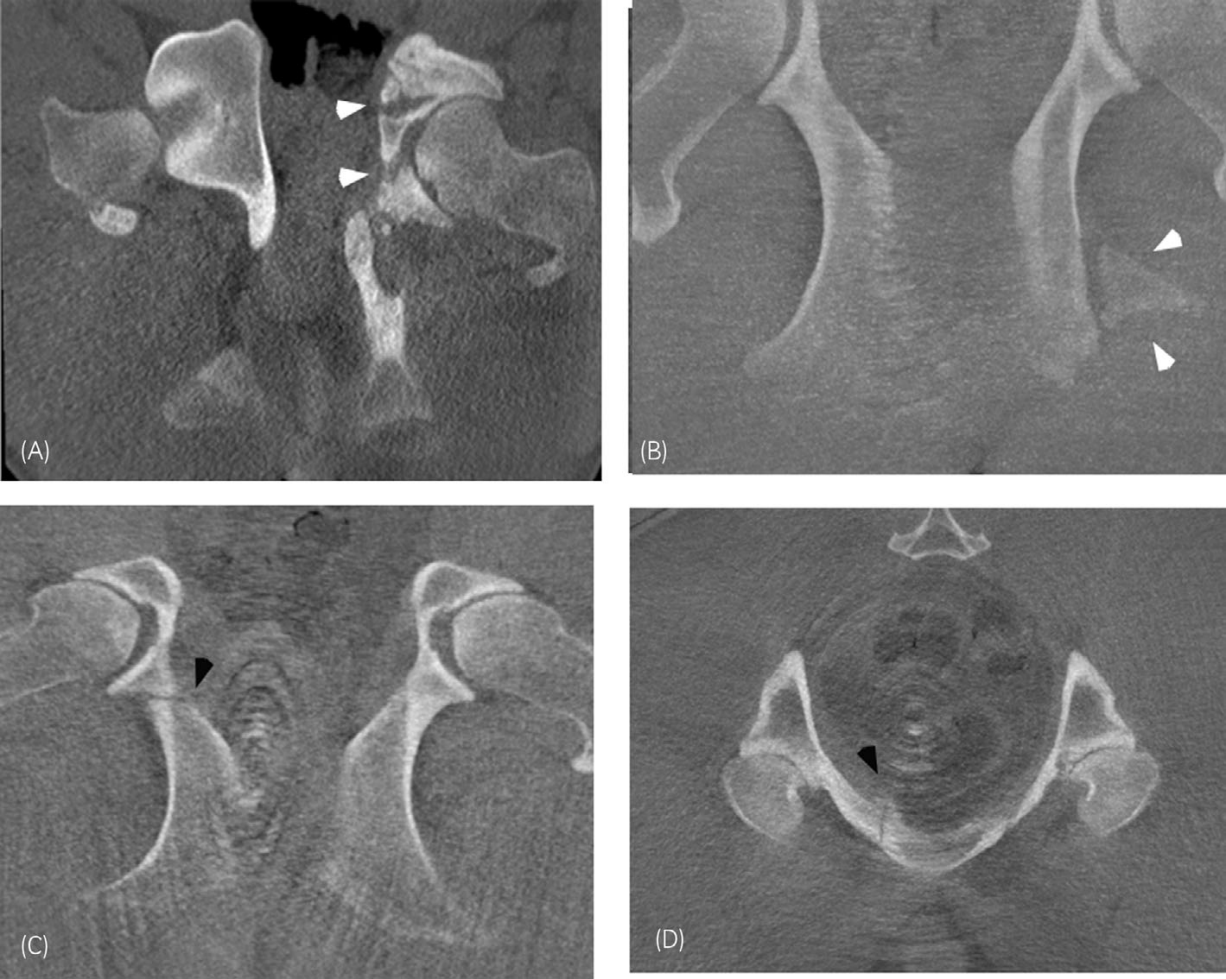
CT images (in bone window) of three horses demonstrating four different pelvic fractures. Left is to the right in all images. Frontal image (A) centred at the level of the coxofemoral joint showing a comminuted pelvic fracture involving the left acetabulum (white arrowheads). Frontal image (B) centred over the caudal pelvis showing a cranially displaced *tuber ischium* fracture (white arrowheads). Frontal (C) and transverse (D) images of the same horse showing a fracture of the ischial body and the pubis (black arrowheads).

Nine horses (9/56, 16.1%, CI 7.6%–28.3%) were diagnosed with fractures of the pelvis (excluding acetabular rim fractures) (Figure 4). The most common pelvis fracture was of the *tuber ischium*, followed by the *tuber coxae*, and other pelvic fractures. Right‐sided fractures were slightly more prevalent (6/9) than left‐sided fractures (3/9). The fractures were considered chronic in all except for one horse which had a traumatic event 1 week before CT examination (hit by a car). There was a history of trauma in most of the horses (6/9).

### 4.11 Tuber ischium fracture

Four horses had fractures involving the *tuber ischium* (5–7 years, median 5.75 and mean 5.5 years), all fractures were comminuted displaced unilateral fractures. In two horses the *tuber ischium* was affected in isolation, and in two horses the *tuber ischium* and the *os ischium* were affected. Two horses presented for lameness, one for unilateral muscle atrophy of the affected limb, and one horse presented for poor performance. In one of the horses with *tuber ischium* and *os ischium* affected, displacement of the *ischium* resulted in an abnormal angle of the acetabulum and osteoarthritis of the coxofemoral joint was present on the same side.

### 4.12 Tuber coxae fracture

Two horses had fractures of the *tuber coxae* (15 and 20 years old). One horse had been diagnosed with a *tuber coxae* fracture 8 months earlier, based on nuclear bone scintigraphy and physical examination, and presented for CT based on persistent lameness. CT confirmed a chronic fracture of the left *tuber coxae* with a large caudally displaced fracture fragment involving the caudal and lateral aspect of the *tuber coxae*, as well as moderate bilateral osteoarthritis of the sacroiliac joints (worse on the left side). The other horse presented for asymmetry of the *tuber coxae*, with CT revealing a small osseous fragment at the left *tuber coxae*, consistent with a small *tuber coxae* fracture, however of greater clinical relevance the horse was also found to have an acetabular rim fracture, osteoarthritis of the coxofemoral joints, and the sacroiliac joints.

### 4.13 Other pelvic fractures

Three horses presented for lameness following trauma had unilateral fractures involving the acetabulum (both 8‐months‐old) and the ilial body (14 years old). One horse had a unilateral acetabular fracture, with a butterfly configuration extending from dorsal to ventral through the body of the ischium at the ischiatic spine. The second horse had a complex unilateral comminuted fracture involving the acetabulum, with an oblique fracture of the ischium from the lateral border of the minor sciatic incisura extending axially and cranially through the lateral ramus of the ischium to the caudal aspect of the acetabulum, and a second articular horizontal fracture from the cranioventral ilial body in a caudal direction to the cranial aspect of the acetabulum. In the third horse, a chronic oblique simple fracture of the right ischial body extending from the dorsocaudal cortex of the ischial body to the cranioventral cortex. A second fracture line was present on the same side, extending from the cranial ramus of the pubis in a caudal direction.

## 5 DISCUSSION

This study shows how CT, performed under general anaesthesia, can be utilised in horses to diagnose a range of caudal spine and pelvic region lesions. The caudal spine and pelvis are commonly reported sites of injury and pain, and numerous reports have highlighted the importance of improved imaging techniques in these areas.^26^, ^27^, ^28^, ^29^, ^30^ The cross‐sectional images produced with CT have enhanced diagnostic ability in regions of complex anatomy such as the head and neck in horses^31^, ^32^, ^33^ and CT is used in small animal and humans with pelvic trauma routinely.^31^, ^34^, ^35^, ^36^, ^37^ We have found similar benefits in the caudal spine and pelvis with CT including less superimposition compared with radiography and the ability to assess numerous sites, some of which are not assessable with other imaging modalities. Notable disadvantages were encountered, some of which detracted from the diagnostic ability, including, beam hardening artefact, noisy images, and poor contrast.^2^ With CT becoming more widely accessible in equine hospitals throughout the United Kingdom, Europe, and elsewhere, CT of the caudal spine and pelvis is likely to increase in popularity and may parallel the widespread adoption of head and neck CT. This case series describes the detection of a range of lesions on CT in the caudal spine and pelvis in a wide range of horse breeds and sizes, with the largest horse included weighing 680 kg.

The most common abnormalities in this study were lesions of the lumbosacroiliac region. This region has a complex anatomy and is commonly implicated as a site of pain in horses with poor performance, hindlimb stiffness, and hindlimb lameness.^18^, ^24^, ^26^, ^30^, ^38^, ^39^, ^40^ Pain in the lumbosacroiliac region can derive from many potential anatomic sources, such as APJs, intervertebral discs/joints, intertransverse joints, lumbosacral joints and sacroiliac joints, and nerves, but the sensory innervation to this region in horses is poorly understood.^5^, ^26^, ^27^, ^28^, ^29^, ^41^, ^42^, ^43^, ^44^ Accurate localisation of pain in this area can be a challenge as sacroiliac joint injections, whether performed with ultrasound guidance or with blind approaches, are considered periarticular.^30^ Studies has showed diffusion of injectate to structures other than the sacroiliac joint including the interosseous sacroiliac ligament, the lumbosacral intertransverse, APJs and the lumbosacral symphysis.^45^, ^46^, ^47^, ^48^ In this case series, CT imaging facilitated the evaluation of these osseous structures and is indicated in the investigation of horses responding positively to diagnostic anaesthesia of the sacroiliac region.

CT identified gross muscle changes in this case series (case 51), but image quality was insufficient to assess the sacroiliac ligaments. A recent study detailed the findings of post‐mortem and histological examinations of horses that had improved clinically following infiltration of the sacroiliac region with local anaesthesia.^49^ Gross pathological changes were not detected in the sacroiliac ligaments or regional muscles, but sciatic or obturator nerve lesions were found in 7/27 horses (26%).^49^ One horse in this case series had abnormalities associated with the sixth lumbar ventral nerve root, detected on *transrectal* ultrasound, yet no abnormalities were evident on CT. It is, therefore, plausible that nerve lesions, and perhaps lesions of the other soft tissue structures including the sacroiliac ligaments, were not detected in the horses undergoing CT. This may explain why lesions were not detected on CT in some horses despite abnormalities on *transrectal* ultrasound of the lumbosacroiliac region. A study in which only 2/11 horses with a clinical diagnosis of sacroiliac joint disease had evidence of sacroiliac joint arthrosis on post‐mortem examination highlights the challenge of detecting all lesions and may indicate that some lumboscaroiliac pain may be secondary.^50^

With all diagnostic imaging, it is important to consider the difference between the imaging findings and the clinical diagnosis. A poor correlation between imaging findings and back pain has been described for over‐riding and impinging dorsal spinous process in horses.^51^, ^52^ In humans, imaging findings are only weakly related to symptoms of lower back pain.^53^, ^54^ Further complicating this interpretation in the lumboscaroiliac region is that few studies describe the normal anatomy of the caudal vertebral column and its anatomical variations,^4^, ^5^, ^6^, ^49^ and there is a poor correlation between pathology and clinical signs.^55^ In a necropsy survey of Thoroughbred racehorses, all had degenerative changes in the sacroiliac joints yet up until the point of death had been deemed sound for racing.^56^ In seven horses in this case series, abnormalities of the lumbosacroiliac region and coxofemoral joint(s) were suspected on ultrasound but abnormalities of the same structures were not identified on CT. Further work is required to establish the most effective imaging modality for the caudal spine and pelvic region.

The clinical significance of ventral spondylosis is unclear in the horse but has been considered subclinical unless inflammation, impingement, or fracture of the osseous proliferation occurs.^55^ In a radiographic study, vertebral body osteophytes were only present in 3% of horses presented for thoracolumbar problems,^57^ however, under‐diagnosis of ventral spondylosis in the lumbar spine is likely due to the limitations of radiography of this region in horses. Further research is needed to determine the clinical significance of lesions detected on CT of the lumbosacroiliac region in horses.

The second most common abnormality in this case series was coxofemoral joint osteoarthritis. Despite osteoarthritis of many other joints being a common cause of poor performance and lameness, osteoarthritis of the coxofemoral joint is rarely described in the horse. A single case report from 1987 describes osteoarthritis in an aged mare diagnosed on antemortem scintigraphy and post‐mortem radiography and post‐mortem examination^58^ and a more recent article describes four horses with coxofemoral joint osteoarthritis diagnosed post‐mortem.^59^ Coxofemoral joint osteoarthritis in horses has been described to manifest as moderate to severe lameness and the tendency for the horse to move on three tracks.^60^ In contrast, we found that horses with bilateral mild osteoarthritis often presented with either mild lameness or poor performance. This is likely a reflection of the ability to identify milder manifestations of coxofemoral joint osteoarthritis with CT compared with radiography and scintigraphy.^61^ Due to the deep location and limited accessibility of the coxofemoral joint, both radiography and ultrasonography have a lower diagnostic yield when it comes to early or mild osteoarthritis.^62^ CT to assess the coxofemoral joint should be considered in lame or poorly performing horses with positive response to diagnostic analgesia of the coxofemoral joint(s), even with negative ultrasonographic and radiographic signs.

Pelvic fractures were infrequently reported in this case series despite being common in racehorses and traumatic fractures occurring in all types of horses. This is likely a reflection of the ability to diagnose many pelvic fractures with traditional imaging modalities.^14^, ^20^, ^62^ In addition, administration of general anaesthesia to a horse with a suspected acute fracture is often contraindicated due to the risk of further displacement.^14^ Except for one horse (with a suspected *tuber ischium* fracture following a traumatic event 1 week earlier), all horses with pelvic fractures in this horse series were chronic and the most common type of fractures was affecting the bony protuberances (*tuber ischium* or *tuber coxae*), lowering the risk associated with general anaesthesia.

In some horses, despite a diagnosis of pelvic fracture being made or suspected before CT, additional information regarding the fracture configuration but also concurrent potential causes of lameness/poor performance were shown on CT. In one horse, with persistent lameness and poor performance and a previously diagnosed chronic *tuber coxae* fracture, moderate bilateral osteoarthritis of the sacroiliac joints (worse on the lame side) was diagnosed. In the other horse with a *tuber coxae* fracture, the horse was found to have an acetabular rim fracture, osteoarthritis of the coxofemoral joints, and the sacroiliac joints. Similarly, an abnormal angle of the acetabulum and osteoarthritis of the coxofemoral joint was diagnosed in one of the horses with a *tuber ischium* fracture. These findings highlight the importance of considering concurrent injuries in horses with traumatic fractures of the pelvis.

The limitations of this study are similar to those of all retrospective studies. Only those case details that were recorded on the clinical notes were available for analysis and not all horses had complete and standardised diagnostic work‐up. The absence of lesions in 15 horses may reflect a limitation of CT or that lesions were not present. The absence of a standardised work‐up hampers this interpretation. Additionally, the study is limited by the population size. Despite the inclusion of 41 horses with lesions considered to be clinically relevant, each subgroup following the classification of lesions was small.

Caudal spine and pelvic CT imaging are likely to increase in popularity as large‐bore CT scanners become more widespread in equine hospitals. CT has been shown to identify several lesion types throughout this anatomical region and aid in the diagnosis of clinical cases. We recommend utilising CT in horses with a diagnosis but where the clinical progression or response to treatment is unexpectedly poor, and in horses where a diagnosis cannot be made with diagnostic anaesthesia, radiography, and ultrasound. As image acquisition times are fast caudal spine and pelvic CT could be performed in horses undergoing appendicular CT that have concurrent clinical signs of lumbosacroiliac pain.

In conclusion, CT of the caudal spine and pelvis can be utilised in horses and ponies for diagnosing a range of clinical disorders which may be causing ‘lumbosacroiliac pain’, poor performance and hindlimb lameness and stiffness. The pathologies we found were diverse and many patients had multiple lesions. Further prospective research is needed to compare standardised clinical, diagnostic analgesia, and diagnostic imaging findings to CT findings. Further work is also required to describe the relationship between congenital, developmental and acquired changes in the caudal spine and pelvis.

## FUNDING INFORMATION

None.

## CONFLICT OF INTEREST STATEMENT

The authors declare no conflict of interest. None of the authors have any financial affiliation with the manufacturer of the CT scanners used in this study.

## AUTHOR CONTRIBUTIONS

**Nadine Kristina Elise Ogden:** Writing – original draft; writing – review and editing; data curation; methodology; conceptualization; formal analysis; software; investigation. **Katja Winderickx:** Conceptualization; writing – review and editing; supervision; resources; investigation. **John David Stack:** Conceptualization; writing – review and editing; supervision; methodology; resources; investigation.

## DATA INTEGRITY STATEMENT

No new data were created or analysed in this study.

## ETHICAL ANIMAL RESEARCH

Research ethics committee oversight not required for this journal: a retrospective analysis of clinical data.

## INFORMED CONSENT

All clients had previously signed informed consent for the use of their horse data in research.

## Supporting information


**Table S1.** Table with case summaries.

**Figure S1.** Flowchart outlining concurrent pathologies. Concurrent pathology was present in seven horses; three horses had concurrent pathology of the lumbosacroiliac region and the coxofemoral joint(s) (yellow boxes), two horses had concurrent pathology of the lumbosacroiliac region and pelvic fracture(s) (green boxes), one horse had pathology of the coxofemoral joint and a pelvic fracture (blue boxes) and finally one horse had pathology of all three regions (pink boxes).

## Data Availability

The data that support the findings of this study are available from the corresponding author upon reasonable request. Open sharing exemption granted by the editor due to lack of provision in the owner consent process.
