# Supplementary material for: Computed tomography of the equine caudal spine and pelvis. Pathological findings in 56 clinical cases (2018–2023)
Source: Equine Vet J. 2024 Oct 20;57(5):1279–89. doi: 10.1111/evj.14426 (PMC12326895; doi:10.1111/evj.14426)
Supplement: Supplementary file 1 — Table S1. Table with case summaries. [file EVJ-57-1279-s001.pdf]

Equine Veterinary Journal BEVA

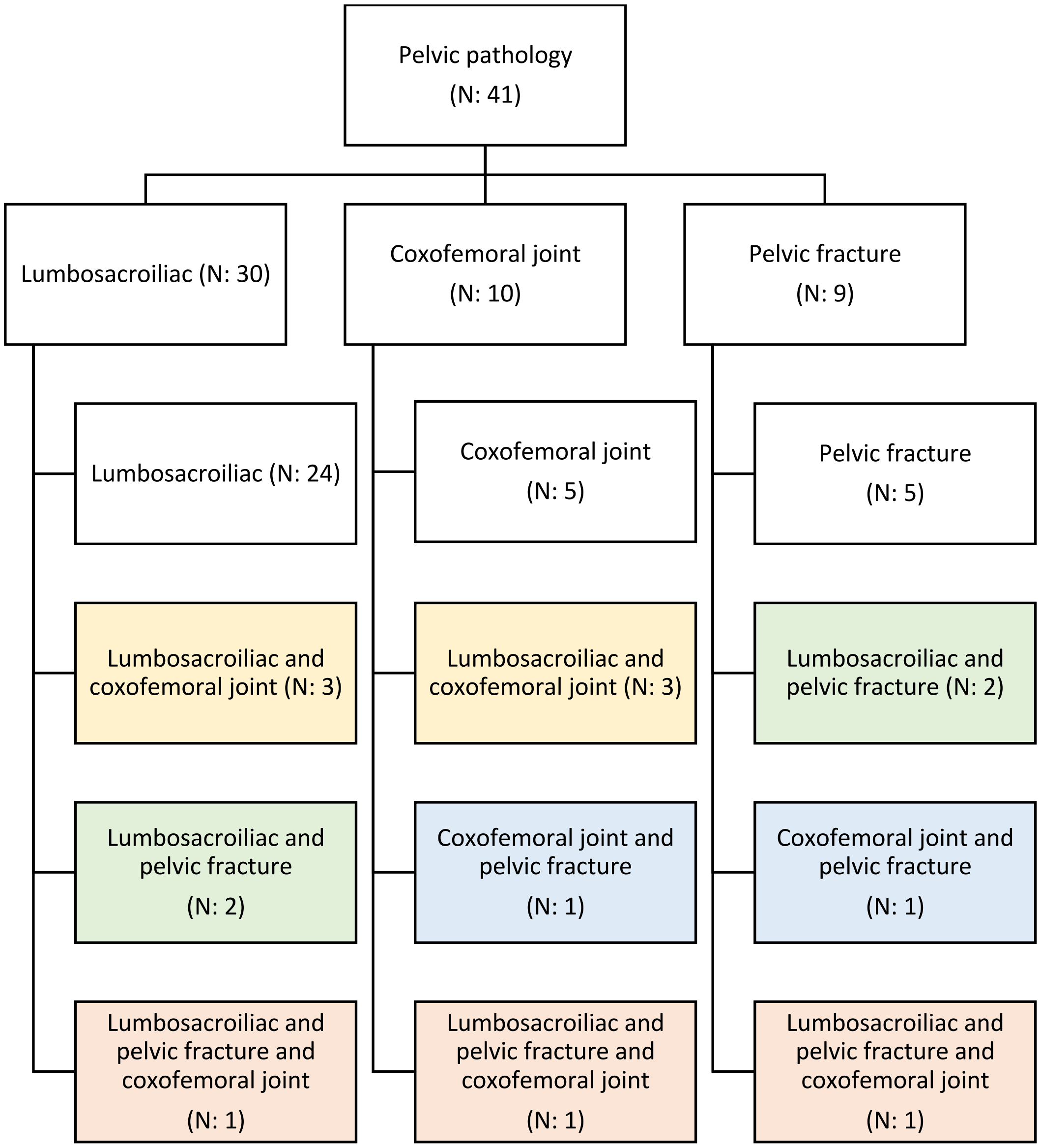

Figure S1: Flowchart outlining concurrent pathologies. Concurrent pathology was present in seven horses; three horses had concurrent pathology of the sacroiliac/lumbosacral region and the coxofemoral joint(s) (yellow boxes), two horses had concurrent pathology of the sacroiliac/lumbosacral region and pelvic fracture(s) (green boxes), one horse had pathology of the coxofemoral joint and a pelvic fracture (blue boxes) and finally one horse had pathology of all three regions (pink boxes).
