# Supplementary material for: Computed tomography of the equine caudal spine and pelvis. Pathological findings in 56 clinical cases (2018–2023)
Source: Equine Vet J. 2024 Oct 20;57(5):1279–89. doi: 10.1111/evj.14426 (PMC12326895; doi:10.1111/evj.14426)
Supplement: Supplementary file 2 — Figure S1. Flowchart outlining concurrent pathologies. Concurrent pathology was present in seven horses; three horses had concurrent pathology of the lumbosacroiliac region and the coxofemoral joint(s) (yellow boxes), two horses had concurrent pathology of the lumbosacroiliac region and pelvic fracture(s) (green boxes), one horse had pathology of the coxofemoral joint and a pelvic fracture (blue boxes) and finally one horse had pathology of all three regions (pink boxes). [file EVJ-57-1279-s002.pdf]

Equine Veterinary Journal BEVA

Table S1: Table with case summaries.

Case number	Breed	Age (years)	Weight (kg)	Presenting complaint	Reason for CT	Diagnosis	Comments
**1**	Icelandic Horse	13	520	Lameness	Lameness investigation: no diagnosis reached with DA/imaging prior to CT	Lumbosacroiliac pathology (sacroiliac joint OA)<br>*Stifle pathology**	Mild lameness, NAD on US/Rx reported by RV<br>DA: neg. to distal limb NB and stifle<br>Scint: IRU SIJ region
**2**	Irish Hunter	11	650	Lameness	Lameness investigation: no diagnosis reached with DA/imaging prior to CT	Lumbosacroiliac pathology (sacroiliac joint OA, intervertebral disc disease, ventral spondylosis)	Mild lameness<br>DA: neg. up to and inc. TMT<br>Rx: stifle NAD<br>US:<br>• stifle abn.<br>• CFJ non-diagnostic<br>• *Transrectal* LSI abn.<br>Scint: IRU SIJ
**3**	Appaloosa	3	530	Lameness	Lameness investigation: no diagnosis reached with DA/imaging prior to CT	Lumbosacroiliac pathology (sacroiliac joint OA)<br>*Stifle pathology**	Bilateral hindlimb lameness<br>DA: neg. up to and incl. tibial and peroneal NB<br>Rx: both HL NAD<br>US: stifle abn.
**4**	Friesian Horse	14	640	Lameness	Lameness investigation: no diagnosis reached with DA/imaging prior to CT	Lumbosacroiliac pathology (sacroiliac joint OA)	Lameness and pyrexia<br>DA: neg. up to and including tib/per nerve block<br>Rx: proximal MT3 abn.<br>US: PSL abn.
**5**	Warmblood /Warmblood cross	14	680	Lameness	Lameness investigation: no diagnosis reached with DA/imaging prior to CT	Lumbosacroiliac pathology (sacroiliac joint OA)<br>CFJ OA	Severe lameness<br>DA: neg. tib/per, neg. stifle<br>Rx: Affected limb NAD<br>US: CFJ abn.<br>Scint: IRU SI and CFJ
**6**	Warmblood /Warmblood cross	9	552	Lameness	Lameness investigation: no diagnosis reached with DA/imaging prior to CT	*Fracture of the central tarsal bone**	DA: neg. distal limb NB, tarsus, stifle<br>RX: tarsus, stifle NAD<br>US: stifle, tarsus, pelvis NAD
**7**	Welsh Pony	6	244	Lameness/trauma	Lameness investigation: no localising imaging findings prior to CT	Lumbosacroiliac pathology (sacroiliac joint OA, intervertebral disc disease, ventral spondylosis)	Very mild lameness<br>Rx: stifles, tarsi NAD<br>US: pelvis NAD
**8**	Friesian Horse	11	550	Lameness	Lameness investigation: localised with DA	Lumbosacroiliac pathology (sacroiliac joint OA, ventral spondylosis)<br>OA of the CFJ(s)	Transient response to IA medication of the CFJ<br>DA; Pos CFJ<br>Rx: affected HL NAD<br>US: CFJ abn.
**9**	Appaloosa	0.66	285	Lameness/trauma	Lameness investigation: localising findings on imaging (US)	Pelvic fracture (other)	Acute lameness after trauma<br>Rx: affected limb and thoracolumbar NAD<br>US;

							• CFJ abn.
**10**	Warmblood /Warmblood cross	0.66	310	Lameness/trauma	Lameness investigation: localising findings on imaging (US)	Pelvic fracture (other)	Traumatic incident in stable<br>Rx; pelvis/CFJ abn.<br>US:<br>• CFJ abn.
**11**	Warmblood /Warmblood cross	14	650	Lameness/trauma	Lameness investigation: localising findings on imaging (US)	Lumbosacroiliac pathology (sacroiliac joint OA)<br>Pelvic fracture (other)	Lameness after a fall<br>Rx: MT2 abn.<br>US:<br>• CFJ abn.
**12**	Shetland Pony	20	204	Lameness	Lameness investigation: localising findings on imaging (US)	OA of the CFJs	Marked lameness<br>US:<br>• CFJ abn.
**13**	Warmblood /Warmblood cross	7	580	Lameness	Lameness investigation: localising findings on imaging (US)	Lumbosacroiliac pathology (sacroiliac joint OA, intervertebral disc disease)	Intermittent lameness<br>Rx: distal limb, tarsus and stifle NAD<br>US:<br>• CFJ abn.<br>• *Transrectal* LSI abn.
**14**	Warmblood /Warmblood cross	20	540	Lameness	Lameness investigation: localising findings on imaging (US)	Lumbosacroiliac pathology (sacroiliac joint OA)<br>OA of the CFJ(s)<br>Acetabular rim fracture(s) Pelvic fracture (*tuber coxae*)	Lameness and asymmetry of tuber coxae<br>US:<br>• CFJ abn<br>• *Transrectal* LSI abn.
**15**	Standardbred Horse	17	570	Lameness	Lameness investigation: localising findings on imaging (US)	Lumbosacroiliac pathology (sacroiliac joint OA, intervertebral disc disease)<br>*Stifle pathology**	Referred for investigations of stifle effusion and *transrectal* US LSI abn.
**16**	Warmblood /Warmblood cross	11	620	Lameness	Lameness investigation: localising findings on imaging (US)	*Stifle pathology**	Painful on SIJ manipulations<br>DA; Mild pos. stifle<br>Rx; stifle NAD, tarsus NAD<br>US:<br>• Stifle NAD<br>• *Transrectal* LSI abn.
**17**	Thoroughbred	10	469	Lameness/trauma	Lameness investigation: localising findings on imaging (US)	Lumbosacroiliac pathology (sacroiliac joint OA)<br>*Proximal suspensory desmitis**	Previous diagnosis of ODSP and PSD, surgical tx for PSD performed following CT under same GA<br>Rx: Thoracolumbar abn.<br>US:<br>• PSL abn.<br>• lumbar APJ abn.
**18**	Warmblood /Warmblood cross	5	550	Lameness	Lameness investigation: localising findings on imaging (US)	*Suspensory branch desmitis**	Mild lameness<br>Rx: affected hindlimb NAD<br>US:<br>• Suspensory ligament branch abn.<br>• CFJ abn.

**19**	Pony (unknown)	6	480	Lameness	Lameness investigation: localising findings on imaging (US)	*Stifle pathology**	Very mild lameness<br>US:<br>• stifle abn.<br>• *Transrectal* LSI abn.
**20**	Thoroughbred	15	526	Lameness/trauma	Lameness investigation: localising findings on imaging (scint.)	Lumbosacroiliac pathology (sacroiliac joint OA, ventral spondylosis)<br>Pelvic fracture (*tuber ischium*)	Scint: diagnosis of TC fx 8m earlier
**21**	Cob	6	380	Lameness/trauma	Lameness investigation: localising findings on imaging (scint.)	Pelvic fracture (*tuber ischium*)	Scint: IRU of TI, lumbar spine and stifle
**22**	Warmblood /Warmblood cross	12	520	Lameness	Lameness investigation: localising findings on imaging (scint.)	*Suspensory branch desmitis**	Previous diagnosed with suspensory branch desmitis, horse has remained lame.<br>DA: pos. low-4-point NB<br>Rx: NAD<br>US: Suspensory ligament branch abn.<br>Scint: IRU of CFJ
**23**	Warmblood /Warmblood cross	6	520	Lameness	Lameness investigation: localising findings on imaging (scint.)	No diagnosis reached**	Mild lameness<br>Scint: IRU CFJ
**24**	Miniature Horse	4	85	Lameness/trauma	Surgical planning/ concurrent pathology	Dislocation of CFJ	Severe lameness, dislocated CFJ
**25**	Shetland Pony	6	138	Lameness/trauma	Surgical planning/ concurrent pathology	Dislocation of CFJ	Severe lameness, dislocated CFJ
**26**	Miniature Horse	5	112	Lameness	Surgical planning/ concurrent pathology	*Lateral luxation of patella**	Severe lameness, dislocated CFJ OA
**27**	Warmblood /Warmblood cross	7	600	Lameness/trauma	Lameness investigation: referred for imaging	Pelvic fracture (*tuber ischium*)	Referred for CT imaging of suspected TI fx
**28**	Warmblood /Warmblood cross	14	530	Lameness	Lameness investigation: referred for imaging	*Stifle pathology**	Referred for imaging, effusion of stifle
**29**	Icelandic Horse	9	315	Lameness	Lameness investigation: further investigation of previous diagnosis/ concurrent pathology	*Proximal suspensory desmitis**	Mild lameness, issues with canter work.<br>DA: pos. to DA of proximal MT region<br>US:<br>• PSL abn.<br>• *Transrectal* LSI NAD
**30**	Friesian Horse	9	650	Lameness	Lameness investigation; unclear indication for CT	US lymphangitis with phlebitis saphenous vein*	PE and US lymphangitis with phlebitis saphenous vein<br>Incomplete medical records relating reason for CT

**31**	Warmblood /Warmblood cross	12	560	Poor performance	Poor performance investigation: localising findings on imaging (US)	Lumbosacroiliac pathology (sacroiliac joint OA)	Lacking propulsion<br>US:<br>• *Transrectal* LSI abn.
**32**	Warmblood /Warmblood cross	3	580	Poor performance	Poor performance investigation: localising findings on imaging (US)	Lumbosacroiliac pathology (sacroiliac joint OA, intervertebral disc disease)	Unable to lunge on the left rein<br>Rx: cervical abn.<br>US:<br>• *Transrectal* LSI abn.
**33**	Warmblood /Warmblood cross	15	520	Poor performance	Poor performance investigation: localising findings on imaging (US)	Lumbosacroiliac pathology (sacroiliac joint OA, intervertebral disc disease)	Issues with canter<br>US:<br>• *Transrectal* LSI abn.
**34**	Warmblood /Warmblood cross	4	536	Poor performance	Poor performance investigation: localising findings on imaging (US)	Lumbosacroiliac pathology (sacroiliac joint OA)	Issues in canter and backing-up<br>Rx: Cervical NAD, thoracolumbar abn.<br>US:<br>• *Transrectal* LSI abn.
**35**	Friesian Horse	5	560	Poor performance	Poor performance investigation: localising findings on imaging (US)	Lumbosacroiliac pathology (L6 ventral nerve root abn)*	Wide hindlimb gait at walk, struggling with canter.<br>US:<br>• *Transrectal* LSI abn.
**36**	Warmblood /Warmblood cross	8	515	Poor performance	Poor performance investigation: localising findings on imaging (US)	Lumbosacroiliac pathology (sacroiliac joint OA)	Issues in canter, painful neck<br>Rx; cervical NAD, thoracolumbar abn.<br>US:<br>• cervical APJ abn.<br>• *Transrectal* LSI abn.
**37**	Warmblood /Warmblood cross Speck Kern	5	540	Poor performance	Poor performance investigation: localising findings on imaging (US)	OA of the CFJ(s)<br>Pelvic fracture (*tuber ischium*)	Rx; cervical spine NAD<br>US:<br>• *Transrectal* LSI abn.<br>• cervical abn.<br>• TI abn.
**38**	Cob	16	465	Poor performance	Poor performance investigation: localising findings on imaging (US)	Lumbosacroiliac pathology (sacroiliac joint OA, intervertebral disc disease, ventral spondylosis)	Problems backing up, mild neurologic signs<br>Rx; cervical NAD<br>US:<br>• Lumbar APJ abn.<br>• cervical NAD
**39**	Warmblood /Warmblood cross	6	620	Poor performance	Poor performance investigation: localising findings on imaging (US)	OA of the CFJ	Wide hindlimb gait, reluctant to collect<br>Rx: stifle and thoracolumbar NAD<br>US:<br>• CFJ abn.<br>• *Transrectal* LSI NAD

**40**	Warmblood /Warmblood cross	5	603	Poor performance	Poor performance investigation: localising findings on imaging (US)	*Stifle pathology**	Difficulty with collection<br>US:<br>• stifle abn.<br>• *Transrectal* LSI abn.
**41**	Arabian Horse	10	500	Poor performance/trauma	Poor performance investigation: localising findings on imaging (Scint)	Lumbosacroiliac pathology (sacroiliac joint OA)<br>OA of the CFJ(s)	History of old pelvic trauma<br>US:<br>• *Transrectal* LSI abn.<br>• PSL abn.<br>Scint: IRU CFJ and SIJ
**42**	Warmblood /Warmblood cross	14	550	Poor performance	Poor performance investigation: localising findings on imaging (Scint)	Lumbosacroiliac pathology (sacroiliac joint OA)	Difficulty with collection, referred for imaging.<br>Scint: IRU SIJ
**43**	Warmblood /Warmblood cross	8	450	Poor performance/trauma	Poor performance investigation: localising findings on imaging (Scint)	OA of the CFJ(s)<br>Acetabular rim fracture(s)	Traumatic incident 2 years previously, performance issues since, not resolved with rest<br>Rx; thoracolumbar abn.<br>US: CFJ abn.<br>Scint: IRU CFJ, DSP thoracic
**44**	Warmblood /Warmblood cross	3	512	Poor performance/trauma	Poor performance investigation: Clinical suspicion of LSI pain	No diagnosis reached**	Traumatic accident 1 month earlier, stiffness and issues with canter<br>Rx: thoracolumbar abn.<br>US:<br>• *Transrectal* LSI NAD
**45**	Welsh Pony	10	350	Poor performance	Poor performance investigation: Clinical suspicion of LSI pain	Lumbosacroiliac pathology (intervertebral disc disease)	Self-mutilation of the rump (biting rump in stable/field and poor performance when ridden). CT also showed changes associated with the intertransverse joints and APJ of possible clinical significance. Following treatment of the SIJ region behaviour resolved.
**46**	New Forest Pony	16	480	Poor performance	Poor performance investigation: Clinical suspicion of LSI pain	Lumbosacroiliac pathology (sacroiliac joint OA, intervertebral disc disease)	Chronic (7y) poor performance and stiffness<br>Rx: cervical and thoracolumbar NAD<br>US: (several years ago)<br>• *Transrectal* LSI NAD
**47**	Thoroughbred	5	512	Poor performance	Poor performance investigation: Clinical suspicion of LSI pain	Lumbosacroiliac pathology (sacroiliac joint OA, intervertebral disc disease)	SI and back pain suspected on PE
**48**	Warmblood /Warmblood cross	6	560	Poor performance	Poor performance investigation: Referred for imaging	Lumbosacroiliac pathology (sacroiliac joint OA)	Bucking, not wanting to go backwards
**49**	Warmblood /Warmblood cross	4	530	Asymmetry: Asymmetric tail carriage	Asymmetry investigation: Referred for imaging	Lumbosacroiliac pathology (sacroiliac joint OA)	Referred for imaging

**50**	Warmblood /Warmblood cross	5	510	Asymmetry: Loss of muscling left pelvic area	Asymmetry investigation: Investigation of muscle atrophy and asymmetry of TI	Pelvic fracture (*tuber ischium*)	Referred for imaging. Asymmetry of TI and muscle loss on affected rump<br>US:<br>• TI abn.
**51**	Warmblood /Warmblood cross	9	385	Asymmetry: Focal muscle atrophy	Asymmetry investigation: Clinical suspicion of pelvic pathology	Lumbosacroiliac pathology (sacroiliac joint OA, intervertebral disc disease)	Marked atrophy of the biceps femoris muscle region on the right side
**52**	Warmblood /Warmblood cross	4	540	Asymmetrical: Asymmetrical croup	Asymmetry investigation: localising findings on imaging (US)	Lumbosacroiliac pathology (sacroiliac joint OA, intervertebral disc disease, ventral spondylosis)	Upwards fixation of the patella, asymmetrical croup<br>Rx: stifle NAD<br>US:<br>• CFJ abn.<br>• *Transrectal* LSI abn.
**53**	Friesian Horse	7	526	Other; Drainage point at sacrum	Other: Investigate drainage tract	Lumbosacroiliac pathology (dorsal dermal sinus)	Drainage point at dorsal sacrum, chronic
**54**	Friesian Horse	4	657	Other: Presented for imaging	Other: Referred for imaging	Lumbosacroiliac pathology (sacroiliac joint OA)	Referred for imaging
**55**	Highland Pony	0.75	250	Other/trauma: Crouched hindlimb gait	Other: Investigation of muscle atrophy	No diagnosis reached**	Fell in a well as a young foal and has had a crouched hindlimb gait since<br>Rx and US: stifles NAD
**56**	Warmblood /Warmblood cross	3	560	Other: Muscle atrophy	Other: Investigation of muscle atrophy	No diagnosis reached**	Atrophy of gluteal muscles 7 weeks port foaling<br>US:<br>• cervical abn.<br>• *Transrectal* LSI NAD

Abbreviations: abnormal (abn.), including (inc.), no abnormalities detected (NAD), negative (neg.), positive (pos.), nerve block (NB), ultrasound (US), radiograph (Rx), computer tomography (CT), increased radiopharmaceutical uptake (IRU), diagnostic anaesthesia (DA), referring veterinarian (RV), proximal suspensory ligament (PSL), third metatarsal bone (MT3), metatarsal (MT), gamma scintigraphy (scint.), articular process joint (APJ), tuber ischium (TI), tuber coxae (TC), sacroiliac joint (SIJ), lumbosacroiliac (LSI), coxofemoral joint (CFJ), osteoarthritis (OA), proximal suspensory desmitis (PSD), over-riding dorsal spinous processes (ORDSP), treatment (tx), fracture (fx).

* Cases with normal pelvis/caudal back CT and a diagnosis reached/aided by CT (under the same general anaesthesia) and/or other imaging modalities in a different anatomical region

** Cases where no diagnosis was reached
